# How the Fibrotic Lung Microenvironment Regulates Stem Cell Behavior: Immune Cues, Extracellular Matrix Remodeling, and Therapeutic Implications

**DOI:** 10.1155/ancp/1319659

**Published:** 2026-01-19

**Authors:** Zihan Zhou, Shuhua Han, Yuanfang Duan, Minqi Hong, Jie Huang

**Affiliations:** ^1^ Department of Respiratory and Critical Care Medicine, Zhongda Hospital, Southeast University, Nanjing, Jiangsu, China, seu.edu.cn; ^2^ Department of Medicine, Southeast University, Nanjing, Jiangsu, China, seu.edu.cn; ^3^ Department of Respiratory and Critical Care Medicine, The First People’s Hospital of Lianyungang, Lianyungang, Jiangsu, China, lygyy.com.cn

**Keywords:** extracellular matrix, inflammatory, microenvironment, pulmonary fibrosis, stem cells

## Abstract

Pulmonary fibrosis (PF) is a chronic and progressive interstitial lung disease characterized by excessive extracellular matrix (ECM) deposition and remodeling of lung structures. Currently, no effective clinical treatments exist to reverse or halt the progression of PF. Consequently, there is an urgent need to identify novel therapeutic strategies for this complex disease. Stem cells, renowned for their self‐renewal and multipotent differentiation capabilities, have garnered considerable attention for their potential therapeutic applications, particularly in regenerative medicine. The primary stem cell types investigated for PF treatment include mesenchymal stem cells (MSCs), induced pluripotent stem cells (iPSCs), and lung progenitor cells. Preclinical studies have demonstrated that these stem cells can reduce inflammation, modulate immune responses, and promote the repair of damaged lung tissue. Although stem cell transplantation shows promise in PF treatment, challenges such as safety, quality control, and therapeutic efficacy remain unresolved. Recent studies have highlighted that stem cells interact with and modify their transplanted environment, influencing their structural properties and chemical composition. These interactions strongly influence stem cell survival, phenotype, and therapeutic efficacy. Understanding these dynamics will inform the development of new strategies to improve the effectiveness of stem cell therapies for PF.

## 1. Introduction

Pulmonary fibrosis (PF) refers to a group of progressive and terminal lung diseases characterized by the replacement of normal lung architecture with disrupted alveolar structures. These structures contain proliferative fibroblasts and myofibroblasts within the extracellular matrix (ECM). Currently, there are no effective treatments available for PF [1]. PF can be classified into two types: those with known causes, such as sarcoidosis, pneumoconiosis, and chronic hypersensitivity pneumonitis, and idiopathic PF (IPF), which is the most common form of lung fibrosis in humans [1, 2]. Prevalence estimates in Asia–Pacific countries range from 0.57 to 4.51 per 10,000 individuals. The average survival postdiagnosis is between 3 and 5 years, although disease trajectories vary [3]. PF leads to irreversible lung function loss due to fibrotic changes, resulting in symptoms such as worsening cough, dyspnea, and reduced quality of life [4]. Lung transplantation remains an option for a minority of patients, while most rely on antifibrotic therapy and supportive care [5, 6]. Despite recent pharmacological advances that slow the decline in lung function, survival rates have not been significantly improved. Untreated IPF typically results in a median survival of 2–3 years [7]. Therefore, the development of safer and more effective treatments remains a major challenge in managing PF.

Stem cells possess unique capabilities of self‐renewal and multipotent differentiation into various cell types, including neurons, adipocytes, chondrocytes, osteoblasts, epithelial cells, and muscle cells [8]. Due to their multipotency, low immunogenicity, and paracrine activities, stem cells have been widely used in treating a variety of conditions, including diabetes, leukemia, autoimmune diseases, neurodegenerative disorders, and both acute and chronic lung injuries [9–13]. Despite their initial promise for PF treatment, significant challenges such as safety, quality control, and therapeutic efficacy must still be addressed.

The cell microenvironment comprises diverse components that shape the conditions surrounding individual cells or cell populations and regulate their behavior through biophysical and biochemical cues. Key elements include the ECM, homotypic and heterotypic neighboring cells, cytokines, hormones, and mechanical forces from tissue movement or fluid flow [14]. Mesenchymal stem cells (MSCs) reside within this dynamic environment, where signals arising from cell–cell interactions, ECM–cell contacts, and transcriptional regulation orchestrate their fate decisions [15]. Recent advances in transcriptomics, particularly single‐cell RNA sequencing (scRNA‐seq) and spatial transcriptomics, have significantly deepened our understanding of the pulmonary microenvironment in fibrosis. These technologies enable high‐resolution characterization of cellular diversity and spatial organization, revealing molecular pathways that shape fibrogenesis [16]. In this review, we outline the key features of the PF microenvironment and discuss how these factors modulate stem cell behavior, and we highlight how this knowledge might be used to refine stem cell–based therapies.

## 2. Microenvironment of PF

Advances in high‐resolution transcriptomic technologies have revealed the complex cellular and extracellular landscape of the fibrotic lung [17]. Building on these insights, Section 2 provides a detailed overview of the major cellular components and ECM characteristics that define the PF microenvironment. By outlining the roles of epithelial cells, fibroblast subsets, immune cells, vascular elements, and matrix alterations, we highlight how these components contribute to inflammation, fibrosis progression, and tissue remodeling.

### 2.1. Epithelial Cells

Repeated exposure of lung epithelial cells to environmental insults disrupts epithelial integrity and promotes airway injury [18]. Damage to alveolar type II (ATII) cells serves as an early trigger in PF, activating macrophages, dendritic cells, and other innate immune cells [19].

Organoid culture and scRNA‐seq have revealed extensive epithelial heterogeneity and uncovered previously unrecognized ATII subpopulations associated with developmental lung disorders and PF. In IPF, classical Epcam^+^/HTII‐280^+^ ATII cells are markedly reduced, accompanied by the emergence of new aberrant epithelial subsets that express transcripts characteristic of conducting airway or ECM–producing cells, while losing genes required for normal ATII function. Vimentin expression and activation of epithelial–mesenchymal transition (EMT) signaling—including TGFBR1, Wnt/β‐catenin, EGFR, PI3K/AKT, ZEB1, SNAI2, and SMAD2/3—are selectively induced in basal and indeterminate epithelial populations. Concurrently, pathways essential for ATII homeostasis such as lipid synthesis and metabolism are suppressed [20].

Basal cells are markedly expanded in interstitial lung diseases (ILDs), with multiple studies demonstrating increases in KRT5^+^, KRT14^+^, and aberrant progenitor‐like cells [21, 22]. Recent scRNA‐seq analyses classify basal cells into classical multipotent basal cells, proliferating basal cells, secretory‐primed basal cells (SPBs), and activated basal cells. CD66^+^KRT5^+^ SPBs are enriched in IPF, suggesting dysregulated basal cell priming as a potential pathogenic mechanism [23, 24]. With the importance of MUC5B and thus secretory airway cells in disease etiology, these observations put forward modulation of basal cell priming as a novel therapeutic strategy in IPF [25, 26].

Collectively, scRNA‐seq studies demonstrate profound remodeling of epithelial composition in ILDs, revealing (1) depletion of normal alveolar epithelial cells, (2) expansion of diverse conducting‐airway‐like cells, and (3) emergence of ILD‐specific transitional cell states that persist in fibrotic lesions.

### 2.2. Fibroblasts

Persistent epithelial injury initiates epigenetic reprogramming, cellular senescence, and secretion of transforming growth factor‐β (TGF‐β), FGFs, cytokines, and procoagulant mediators, driving fibroblast activation and promoting myofibroblast expansion.

Our research team previously employed scRNA‐Seq technology to categorize fibroblasts into seven distinct subclusters based on known fibroblast markers: inflammatory fibroblasts, resting fibroblasts (two types), myofibroblasts, germ1^high^ fibroblasts, ECM‐fibroblasts, and Lipofibroblasts. Notably, germ1^high^ fibroblasts are predominantly expressed in focal regions within spatial maps. The top 50 genes expressed in germ1^high^ fibroblasts overlap with genes found in inflammatory fibroblasts, ECM fibroblasts, and myofibroblasts, Gene Ontology analyses reveal enrichment in cell adhesion, inflammatory signaling, apoptosis regulation, and myofibroblast differentiation. Spatial transcriptomics and immunohistochemistry confirm Grem1 expression in lesion‐associated fibroblasts, supporting its potential as an early biomarker of fibrosis [27]. Other spatial transcriptomic studies have further characterized fibroblast‐associated gene signatures in IPF, identifying upregulated genes such as FNDC1, COL10A1, and THY1 enriched around fibroblastic foci—regions tightly linked to disease activity. Aberrant epithelial populations (e.g., KRT5^−^/KRT17^+^ cells) frequently localize near fibroblast clusters, supporting a reciprocal epithelial–mesenchymal crosstalk that drives fibrotic progression [17].

### 2.3. Immune Cells

Following ATII injury, dysregulated inflammatory responses contribute to persistent immune activation and chronic fibrotic remodeling [28].

Macrophages are central regulators of these processes. scRNA‐seq studies have identified distinct macrophage states enriched in PF, including PPARG^+^MRC1^+^MARCO^+^ alveolar macrophage subsets with reduced APOE and MAFB expression and transcriptional programs associated with secretion, ECM remodeling, and cell migration [29]. Other studies describe expansion of monocyte‐derived SPP1^+^ macrophages—marked by genes such as MAFB, CD163, and LGMN—which accumulate in fibrotic tissues across multiple datasets [30, 31].

Beyond macrophages, other immune cells contribute to PF pathogenesis. Neutrophils promote fibrosis in bleomycin‐induced models, though their role in human PF requires further validation [32]. NK cells and B cells are reportedly more abundant in nonfibrotic regions of diseased lungs, whereas regulatory T cells appear increased in IPF [26, 30]. Nevertheless, lymphocyte contributions remain incompletely understood [33], with much of the current knowledge derived from preclinical models, which may not fully capture the complexity of the disease in humans.

### 2.4. ECM

The ECM provides structural integrity, mechanical stability, and elastic recoil essential for normal lung function [34]. It also serves as a reservoir for growth factors and cytokines that regulate epithelial proliferation, migration, tissue remodeling, and apoptosis. In healthy lungs, ECM fibers are well organized and basement membranes remain intact. Following injury, activated myofibroblasts deposit large quantities of collagen and ECM proteins [35]. In PF, excessive deposition leads to disorganized fibers, disrupted basement membranes, and stiffened interstitial spaces [36, 37]. Fibrocytes—precursors of collagen‐producing fibroblasts—accumulate in active fibrotic regions, while fibroblasts themselves secrete chemotactic mediators that recruit inflammatory cells [34].

The ECM profoundly shapes immune function through biochemical, structural, and mechanical cues [38]. Altered ECM components, including collagens, hyaluronan, glycosaminoglycans, and proteoglycans, signal via receptors such as CD44 to regulate leukocyte retention and activation [38, 39]. Mechanical stiffening further modulates immune responses: monocyte‐derived macrophages sensing cyclical stretch activate a Piezo1‐HIF1α inflammatory program essential for fibrosis [40], while mechanical stress enhances TGF‐β activation, driving fibroblast differentiation and ECM deposition [41]. Matrix metalloproteinases (MMPs)—normally tightly regulated—are dysregulated in PF, with increases in MMP1, MMP3, MMP7, MMP8, and MMP9 and reductions in antifibrotic MMP13 and MMP19 [42].

These microenvironmental abnormalities—which impair cell survival, engraftment, and regenerative signaling—may underlie the inconsistent and often modest therapeutic responses seen in current clinical trials of stem cell therapy for PF.

## 3. Clinical Evidence, Safety Concerns, and Current Limitations of Stem Cell Therapy in PF

Clinical studies investigating MSC–based or pluripotent stem cell–derived therapies for PF remain limited and have produced inconsistent efficacy outcomes. Most trials were early‐phase studies with small sample sizes, short follow‐up periods, and lacked placebo control, making it difficult to draw firm conclusions regarding therapeutic benefit. Several studies reported stabilization of lung function in a subset of patients, whereas others demonstrated continued clinical decline despite treatment, highlighting the uncertain and possibly limited efficacy of current stem cell approaches [43, 44].

Safety remains a major concern, and the key differences in efficacy, safety profiles, and translational challenges among MSCs, embryonic stem cells, and induced pluripotent stem cell–derived products are summarized in Table 1. Although MSCs are generally well tolerated, infusion‐related reactions, infections, and rare cases of malignancy have been reported. Autologous MSCs from IPF patients may exhibit chromosomal abnormalities or cellular senescence, raising questions about their suitability. Pluripotent stem cell–derived products (ESCs and induced pluripotent stem cells [iPSCs]) carry inherent risks of tumorigenicity, immune activation, and genetic instability. A central challenge across all cell types is poor survival, engraftment, and retention of transplanted cells in the fibrotic lung [45, 46]. Technical and clinical hurdles further limit translation. Optimal delivery route, dose, timing, and frequency of administration remain undefined, and different routes carry distinct risks—from pulmonary embolism after intravenous infusion to airway irritation with local delivery. Variability in cell source, donor age, manufacturing quality, and expansion protocols complicates standardization. High costs, limited specialized infrastructure, and unresolved ethical issues (especially for ESC–based therapies) hinder widespread application [43–45]. Although preclinical data are encouraging, stem cell therapy for PF is still at an early stage, and key issues regarding long‐term safety, efficacy, and feasibility in routine clinical practice remain unresolved. More rigorous, large‐scale, and controlled clinical trials are needed to determine which cell type, dosing strategy, and delivery approach can produce reliable therapeutic benefit.

**Table 1:** Comparative overview of MSCs, iPSCs, and lung progenitor cells in PF therapy.

Cell type	Major advantages	Key limitations	Potential applications in PF
MSCs	Strong immunomodulatory activity; anti‐inflammatory and antifibrotic effects; generally favorable safety profile; readily accessible	High heterogeneity; function influenced by donor age and disease status; poor survival and retention after lung delivery	May attenuate inflammation and modify the microenvironment, but limited capacity for structural regeneration
iPSCs	Robust self‐renewal; ability to differentiate into lung‐relevant lineages; scalable manufacturing	Tumorigenicity risk; genomic/epigenetic instability; high production and regulatory burden	Potential for cell replacement and lung tissue engineering, but clinical translation remains early stage
LPCs	Lung‐specific regenerative potential; ability to restore epithelial architecture; theoretically more targeted	Difficult to obtain; limited expansion capacity; minimal clinical experience	May promote epithelial repair, yet applications remain exploratory

Abbreviations: iPSCs, induced pluripotent stem cells; LPCs, lung progenitor cells; MSCs, mesenchymal stem cells.

Importantly, the inconsistent therapeutic outcomes observed in clinical studies suggest that factors beyond cell type and delivery strategy may fundamentally constrain the efficacy of stem cell–based interventions. Increasing evidence indicates that the severely altered inflammatory and fibrotic microenvironment of the diseased lung—characterized by persistent immune activation, excessive matrix remodeling, oxidative stress, and aberrant cytokine signaling—may impair transplanted cell survival, homing, differentiation, and reparative function. These microenvironmental abnormalities not only limit the therapeutic potential of administered cells but may actively reprogramme or exhaust them after transplantation. Therefore, a deeper understanding of how the PF microenvironment shapes stem cell behavior has become essential for explaining the limited efficacy observed to date and for guiding the development of next‐generation, microenvironment‐responsive stem cell therapies.

## 4. The Impact of the Inflammatory Microenvironment on Stem Cell Biology and Therapeutic Potential

The inflammatory microenvironment, together with immune dysregulation, critically shapes stem cell behavior through a complex cytokine and chemokine network. During the early inflammatory phase of PF, key cytokines such as tumor necrosis factor‐α (TNF‐α), interleukin (IL)‐1β, and IL‐6 are rapidly induced and modulate stem cell proliferation, differentiation, migration, and survival [47]. Persistent inflammation, oxidative stress, and immune imbalance further drive ECM remodeling, thereby altering stem cell niches and impairing reparative functions. Understanding how inflammatory cues reprogramme stem cell fate, homing, and paracrine responses is essential for optimizing cell‐based therapies in PF, including the design of preconditioning strategies and the selection of appropriate therapeutic windows.

### 4.1. Effect of the Inflammatory Microenvironment on Stem Cell Proliferation and Apoptosis

The capacity for self‐renewal and controlled proliferation is fundamental for stem cell–mediated tissue repair. Intrinsic pathways such as Wnt, Notch, and Hedgehog regulate gene expression and cell fate, but their activity is strongly influenced by extrinsic inflammatory cues [48]. In the injured lung, transient increases in IL‐1β and TNF‐α within the alveolar niche can support epithelial regeneration. Using an organoid‐based screening assay, Katsura et al. [49] showed that influenza‐induced injury upregulates IL‐1β and TNF‐α in the ATII epithelial cell (AEC2) niche, and that NF‐κB‐dependent signaling maintains the differentiation potential and enhances the proliferation of surviving AEC2s, thereby promoting lung regeneration.

Chronic or noxious exposures, however, often exert more complex and sometimes paradoxical effects. Long‐term cigarette smoke (CS) exposure induces persistent inflammation, oxidative stress, and epithelial apoptosis, ultimately degrading the lung matrix. Tsutsumi et al. [50] reported that CS enhances the “stemness” of ATII cells, increasing their proliferation, differentiation, and resistance to apoptosis compared with air‐exposed controls, suggesting a maladaptive expansion of progenitors under chronic stress. Oxidative stress can also directly compromise airway stem cells. Liu et al. [51] found that H_2_O_2_–induced oxidative stress significantly increased apoptosis and necrosis in airway basal stem cells, whereas pretreatment with coenzyme Q10 (CoQ10) reduced oxidative damage and improved cell survival after transplantation, highlighting the value of antioxidant conditioning.

Systemic or recurrent inflammation may exhaust stem cell pools and accelerate functional aging. Bogeska et al. [52] observed irreversible depletion of functional hematopoietic stem cells (HSCs) following repeated inflammatory or infectious challenges, with persistent signatures of accelerated aging in challenged mice. Similarly, Mitchell et al. [53] showed that chronic IL‐1β production by endosteal stromal cells leads to inflammatory remodeling of mesenchymal stem and progenitor cells, depletion of endosteal mesenchymal populations, impaired osteogenesis, and vascular dysfunction in the bone marrow niche.

Beyond animal models, patient‐derived inflammatory fluids provide relevant insights for PF–related therapies. Bronchoalveolar lavage fluid (BALF) and serum from individuals with inflammatory lung diseases have been used to model the diseased milieu. In cystic fibrosis (CF), BALF containing fungal‐derived gliotoxin triggers rapid MSC apoptosis by disrupting mitochondrial function. Transcriptomic profiling of MSCs exposed to aspergillus‐positive versus aspergillus‐negative CF BALF revealed differential expression of genes involved in interferon signaling, antimicrobial responses, and cell death programs [54]. Together, these data show that inflammatory and oxidative cues can transiently support progenitor expansion but, when chronic or excessive, drive stem cell exhaustion and apoptosis—an issue that should be considered when designing cell therapies for PF.

### 4.2. Effect of the Inflammatory Microenvironment on Stem Cell Migration and Homing

Stem cell migration and homing are tightly regulated processes that enable cells to reach injured regions and exert reparative functions. This behavior is governed by inflammatory cytokines, chemokines, adhesion molecules, and growth factors. TNF‐α, a prototypical pro‐inflammatory cytokine frequently elevated in inflamed lung tissue, has a pronounced impact on MSC motility [55]. Preconditioning rat MSCs with TNF‐α enhances their migratory capacity via increased phosphorylation of NF‐κB‐p65 and upregulation of the chemokine receptor CXCR4 on the cell surface [56]. At higher concentrations, TNF‐α also modulates epitranscriptomic regulation. Xie et al. [57] showed that TNF‐α reduces N6‐methyladenosine (m6A) modifications in human bone‐marrow MSCs, stabilizing engulfment and cell motility protein 1 (ELMO1). ELMO1 then interacts with dedicator of cytokinesis 1 (DOCK1) to activate Rac1, further promoting MSC migration. IL‐1β exerts similar effects by enhancing MSC migration and adhesion and facilitating leukocyte chemotaxis through NF‐κB‐dependent secretion of soluble mediators [58].

Chemokine–receptor axes are central to directing stem cell trafficking. The stromal cell–derived factor‐1 (SDF‐1/CXCL12)/CXCR4 pathway is particularly important for MSC homing toward inflamed lung tissue. Under inflammatory conditions, CXCL12 is upregulated in damaged niches and guides CXCR4‐positive MSCs to sites of injury [59]. In line with this, pre‐exposure of MSCs to TGF‐β has been shown to enhance homing by upregulating CXCR4, whereas pharmacological inhibition of TGF‐β receptor II or CXCR4 diminishes this effect [60]. MSCs also respond dose‐dependently to CXCL12 and CXCL8 (IL‐8); blocking CXCR2 with neutralizing antibodies reduces bone marrow‐MSC migration, highlighting a complementary role of the IL‐8/CXCR2 axis in chemotaxis [61].

Innate immune cells, particularly macrophages, further sculpt stem cell migratory behavior through cell–cell interactions and cytokine crosstalk. Macrophage‐derived chemokines such as CXCL10 and CCL5, as well as macrophage migration inhibitory factor (MIF), promote MSC migration and invasion, partly via activation of the MAPK and PI3K/AKT pathways through CXCR2 and CXCR4 [62, 63]. In the context of PF, these observations suggest that carefully tuned inflammatory preconditioning and targeted manipulation of chemokine pathways may improve the homing efficiency of therapeutic stem cells to fibrotic lung regions.

### 4.3. Effect of Inflammatory Microenvironment on Stem Cell Differentiation

MSCs possess broad differentiation potential, giving rise to osteogenic, adipogenic, and chondrogenic lineages—properties frequently used as criteria for “stemness.” However, inflammatory cues can profoundly reshape lineage commitment. TNF‐α has emerged as a key pro‐inflammatory cytokine that consistently inhibits osteogenic differentiation of MSCs through multiple mechanisms. In postmenopausal osteoporosis, TNF‐α‐induced upregulation of SOX5 suppresses osteogenesis of human MSCs via the Kruppel‐like factor 4 (KLF4) signaling pathway [64]. TNF‐α also increases the expression of P2Y2 purinoceptor through ERK and JNK activation; silencing P2Y2 partially rescues TNF‐α‐mediated inhibition of MSC proliferation and osteogenic differentiation [65]. In human periodontal ligament stem cells, TNF‐α reduces alkaline phosphatase activity and downregulates osteogenesis‐related genes such as RUNX2 and osteocalcin (OCN) under osteogenic conditions [66]. Moreover, TNF‐α has been reported to impair osteogenesis through aberrant activation of canonical Wnt signaling [67].

Beyond direct cytokine‐mediated effects, immune cells within the inflammatory microenvironment also regulate stem cell differentiation. Huang et al. [68] found that punicalagin (PUN) modulates the immune microenvironment by promoting M2 macrophage polarization and reducing oxidative stress via activation of the nuclear factor erythroid 2‐related factor 2 (Nrf2)/heme oxygenase‐1 (HO‐1) pathway. These effects enhance the osteogenic differentiation of BMSCs and stimulate angiogenesis in human umbilical vein endothelial cells (HUVECs). Taken together, these studies suggest that pro‐inflammatory cytokines such as TNF‐α tend to divert MSCs from osteogenic differentiation, whereas strategies that promote anti‐inflammatory or pro‐regenerative immune profiles can partially restore their osteogenic capacity. In PF, where chronic inflammation and aberrant macrophage activation coexist, modulating inflammatory signaling and macrophage polarization may help preserve or re‐establish the reparative potential of resident or transplanted stem cells.

### 4.4. Effect of the Inflammatory Microenvironment on Stem Cell Paracrine and Immunomodulatory Functions

Stem cells exert much of their therapeutic activity through the secretion of bioactive factors that modulate surrounding immune and structural cells. In inflammatory settings, the paracrine and immunomodulatory profiles of MSCs are dynamically regulated, leading to either amplification or resolution of inflammation. Proteomic analyses of human and murine MSC–conditioned media have shown that exposure to pro‐inflammatory cytokines enhances the secretion of immunoregulatory and angiogenic proteins in both species [69]. BALF from patients with acute respiratory distress syndrome (ARDS) or healthy controls does not necessarily induce cytotoxicity in MSCs but can significantly alter their expression of pro‐ and anti‐inflammatory mediators. Notably, IL‐1β levels in both healthy and ARDS BALF predict MSC production of cytokines such as IL‐6 and IL‐8, underscoring the sensitivity of MSC secretomes to inflammatory cues [70].

TNF‐α is among the earliest cytokines released during inflammation and can either exacerbate or restrain the inflammatory response, depending on context and dose. Pre‐stimulation of human bone marrow–derived MSCs (hBMSCs) with TNF‐α upregulates cyclooxygenase‐2 (COX‐2) and PGE2. When co‐cultured with macrophages, these preconditioned MSCs promote IL‐10 release from M2 macrophages while reducing TNF‐α levels in the culture medium, indicating enhanced anti‐inflammatory capacity [71]. TNF‐α also induces the expression of tumor necrosis factor‐α‐stimulated gene‐6 (TSG‐6), a pivotal anti‐inflammatory mediator produced by MSCs. Treatment with TNF‐α increases TSG‐6 secretion in hBMSCs and human induced pluripotent stem cell‐derived MSCs (iPSC‐MSCs), where TSG‐6‐dependent pathways suppress inflammation in an Akt‐dependent manner and promote epithelial proliferation, accelerating mucosal healing in a colitis model [72, 73]. Interactions with activated innate and adaptive immune cells further refine MSC paracrine outputs. Coculture with T cells increases MSC secretion of growth factors such as epidermal growth factor (EGF), platelet‐derived growth factor‐AB/BB (PDGF‐AB/BB), fibroblast growth factor‐2 (FGF‐2), and vascular endothelial growth factor (VEGF), while contact with NK cells stimulates transforming growth factor‐α (TGF‐α) production [74]. These observations highlight that the inflammatory microenvironment is a double‐edged sword: while excessive inflammation can damage or deplete stem cells, controlled inflammatory preactivation can “license” MSCs to produce a more potent immunoregulatory and pro‐regenerative secretome. In PF, carefully exploiting this “licensing” effect—while limiting overt cytotoxicity—may improve the design of MSC–based interventions.

## 5. Effect of the ECM on Stem Cells

In fibrotic lungs, changes in the biochemical composition and mechanical properties of the ECM strongly affect how stem cells sense and respond to their niche. Stem cells interpret these cues mainly through integrins and other adhesion receptors that engage ECM components to activate signaling pathways controlling proliferation, survival, migration, differentiation, and senescence [75]. Under physiological conditions, such interactions maintain a balanced regenerative niche. In PF, however, disrupted mechanotransduction and aberrant matrix stiffness convert these signals into drivers of maladaptive activation or exhaustion, impairing endogenous repair and diminishing the efficacy of stem cell–based therapies [76].

### 5.1. Effect of ECM Proteins on Stem Cell

ECM proteins such as collagen, fibronectin, laminin, and elastin provide both structural support and instructive cues that shape stem cell behavior. Collagen, the major structural ECM component, is essential for mechanical integrity and tissue regeneration. However, pathological modifications can impair its capacity to support stem cells. Glycated collagen, for example, markedly alters the morphology of adipose‐derived MSCs, reducing proliferation, focal adhesion formation, and actin cytoskeletal organization, ultimately compromising their function [77].

Tropoelastin, the soluble precursor of elastin, assembles into elastic fibers that confer resilience to tissues but also exhibits cell‐instructive properties. Recent work has shown that tropoelastin exerts strong mitogenic effects on HSCs and MSCs. Transcriptomic analyses indicate that tropoelastin enhances MSC proliferation by upregulating cell‐cycle genes while simultaneously downregulating cell‐cycle inhibitors and genes associated with senescence initiation and progression, thereby expanding MSC populations and delaying cellular aging [78, 79].

MMPs, a family of zinc‐dependent endopeptidases, are key regulators of ECM remodeling due to their ability to degrade collagen, elastin, laminin, and fibronectin [80]. Ries et al. [81] reported that BMSCs express high levels of MMP‐2, membrane‐type 1 MMP (MT1‐MMP), and their endogenous inhibitors TIMP‐1 and TIMP‐2. Silencing MMP‐2, MT1‐MMP, or TIMP‐2 impairs MSC migration, whereas TIMP‐1 knockdown enhances migratory capacity, indicating that a finely balanced MMP/TIMP axis is required for effective MSC trafficking. Recombinant human heat shock protein 90‐α (rhHsp90α) further promotes MSC migration by upregulating MMP‐2 and MMP‐9 expression, secretion, and enzymatic activity via PI3K/AKT and ERK pathways [82]. In PF, where ECM composition is profoundly altered, dysregulation of these protease systems may either hinder appropriate stem cell recruitment or contribute to pathological remodeling, suggesting that targeted modulation of ECM protein dynamics could improve stem cell–based therapies.

### 5.2. Effect of ECM Mechanical Properties on Stem Cells

#### 5.2.1. Biomechanical Properties

The biomechanical properties of the ECM, particularly stiffness and micro‐topography, play pivotal roles in regulating stem cell fate. Studies manipulating hydrogel stiffness to mimic different ECM conditions have shown that mechanical cues can redirect lineage commitment and reshape cellular metabolism. Yue et al. [83] demonstrated that rigid hydrogels (39–45 kPa) upregulate aminoacyl‐tRNA biosynthesis and favor osteogenesis, whereas softer hydrogels (7–10 kPa) enhance unsaturated fatty acid biosynthesis and glycosaminoglycan deposition, promoting adipogenic and chondrogenic differentiation of BMSCs. Similarly, Fu et al. [84] used elastomeric micropost arrays to independently tune substrate stiffness and found that stiff substrates favor osteogenic differentiation of hMSCs, while softer ones promote adipogenesis.

Mechanical cues also influence nuclear organization and gene regulation. Na et al. [85] used high‐throughput chromosome conformation capture to show that MSCs cultured on stiff ECM exhibit increased long‐range chromatin interactions and alterations in A/B compartmentation and topologically associating domains (TADs), affecting genes involved in cytoskeletal organization and osteogenesis. ECM stiffness further modulates the production of extracellular vesicles (EVs). MSCs seeded on soft tissue‐mimetic hydrogels with low integrin ligand density secrete approximately 10 times more EVs per cell than those on rigid plastic, without loss of therapeutic efficacy in acute lung injury models. Enhanced transport and fusion of CD63^+^ multivesicular bodies with the plasma membrane underlie this increased EV release, whereas the Arp2/3 complex limits MVB trafficking and EV secretion on hydrogels [86]. Conversely, reduced ECM stiffness can disrupt the sorting of vesicular transport‐related proteins and autophagy‐related lipids into MSC–derived EVs, impairing their secretion and uptake by macrophages [87].

Substrate stiffness acts in concert with surface topography and cell shape to influence proliferation and lineage specification. For example, pillar‐patterned substrates promote large, spherical MSC morphologies, while stiffness remains the dominant determinant of osteogenic versus neuronal differentiation [88]. In mouse embryonic stem cells, substrate stiffness is critical for maintaining stemness, whereas distinct topographies modulate colony morphology, from flattened to spheroid forms [89]. Cell shape itself is a key determinant of fate: McBeath et al. [90] showed that spread, well‐adherent hMSCs preferentially undergo osteogenesis, whereas rounded and poorly spread cells tend toward adipogenesis via RhoA‐ROCK signaling. Together, these data indicate that ECM stiffness, microarchitecture, and cell geometry cooperate to direct stem cell fate—factors that are markedly altered in fibrotic lungs.

#### 5.2.2. Mechanotransduction

Mechanical signals generated by the ECM are converted into biochemical responses through mechanosensitive ion channels, cytoskeletal networks, and force‐responsive transcriptional regulators. Piezo channels are prototypical mechanosensitive ion channels activated by membrane stretch, shear stress, substrate stiffness, tissue compression, and osmotic changes. Their activation increases Ca^2+^ influx, which can promote proliferation via ERK phosphorylation and modulate differentiation through Notch‐dependent mechanisms, depending on cellular context [91–93].

Mechanical forces transmitted through the cytoskeleton also regulate the nuclear localization and activity of transcriptional coactivators. Panciera et al. [94] showed that ECM stiffness directly controls the nuclear accumulation of yes‐associated protein (YAP) and transcriptional coactivator with PDZ‐binding motif (TAZ); higher stiffness promotes nuclear YAP/TAZ and correlates with increased proliferation and migration, whereas softer matrices retain YAP/TAZ in the cytoplasm. Silver et al. [95] similarly observed that elevated ECM stiffness enhances muscle stem cell migration and proliferation by facilitating YAP/TAZ nuclear translocation.

Actin cytoskeleton remodeling contributes to mechanotransduction by altering nuclear G‐actin levels. Depolymerization of actin generates G‐actin that translocates into the nucleus, where it interacts with the serum response factor (SRF) cofactor MAL, leading to SRF activation and induction of immediate‐early genes such as Fos and JunB [96–98]. AP‐1 transcription factors formed by these gene products drive cell cycle progression through upregulation of cyclin‐dependent kinases and cyclin D1 [99].

#### 5.2.3. Surface Properties

Integrins are central mechanosensors that couple ECM composition and mechanics to cytoskeletal dynamics and downstream signaling [100]. They regulate cell adhesion, spreading, traction forces, and the activation of pathways such as FAK/Src, RhoA‐ROCK, and PI3K/AKT. Lu et al. [101] identified integrin β1 (Intgβ1) as an essential subunit in iPSCs, necessary for adhesion to endothelial cells; genetic ablation or siRNA‐mediated knockdown of Intgβ1 markedly impaired iPSC adhesion and migration across activated endothelial monolayers. In line with this, Ip et al. [102] reported that blocking integrin β1 reduces bone‐marrow MSC migration into infarcted myocardium, emphasizing the importance of ECM–integrin interactions for efficient stem cell homing.

Beyond cell adhesion, the ECM serves as a reservoir for growth factors that further modulate stem cell behavior. ECM–modified decellularized tendon slices (ECM‐DTSs) have been shown to enhance BMSC migration via activation of the PI3K/AKT pathway [103]. Basic fibroblast growth factor (bFGF), sequestered within ECM niches, promotes MSC migration through the Akt/PKB signaling pathway [104]. These findings illustrate how ECM composition, mechanical properties, and surface ligands are integrated through integrin‐mediated mechanosensing and growth factor presentation to regulate stem cell adhesion, migration, fate decisions, and paracrine activity. In fibrotic lungs, strategies aimed at normalizing ECM properties or engineering PF‐mimetic yet “reprogrammable” matrices may help restore a more favorable niche for both endogenous and transplanted stem cells.

## 6. Conclusions and Future Prospects

**Figure 1:**
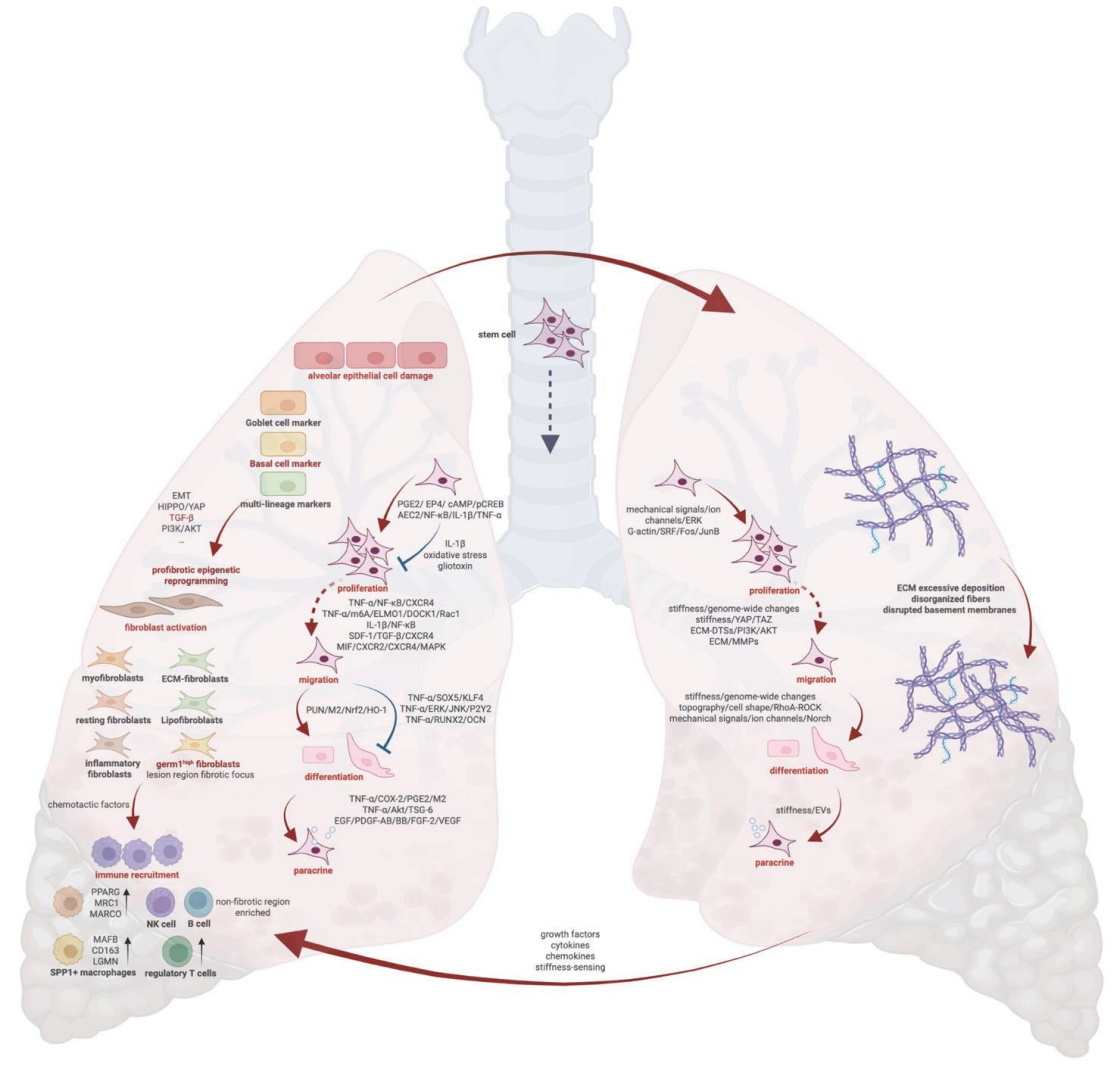
An integrative schematic summary of the review.

Cell‐based therapies are being explored as an adjunct to pharmacological and surgical treatments in many diseases, including PF [105]. Owing to their self‐renewal capacity and tissue‐repair potential, stem cells are attractive candidates for tackling complex and refractory conditions [106]. However, the therapeutic efficacy of stem cell–based interventions is profoundly shaped by the local microenvironment into which the cells are delivered. This review synthesizes current knowledge on how alveolar epithelial injury, fibroblast activation, immune dysregulation, and extracellular matrix remodeling collectively influence the survival, phenotype, and reparative functions of transplanted stem cells. A schematic summary of these interactions is presented in Figure 1. Despite significant progress, major gaps remain in understanding the in vivo fate of infused MSCs—including their engraftment efficiency, phenotypic stability, and functional persistence within the fibrotic niche. Furthermore, clinical trials investigating stem cell therapy for PF are still in early developmental stages and remain limited in scope.

Looking ahead, future research should prioritize technologies that enable precise manipulation and modeling of the PF microenvironment. Biomaterial‐assisted delivery systems, such as injectable hydrogels and microporous scaffolds, offer the potential to modulate critical microenvironmental parameters—including matrix stiffness, oxygen availability, and cytokine exposure—to enhance stem cell retention and reparative activity in vivo [107, 108]. Gene editing technologies such as CRISPR/Cas9 may allow the engineering of stem cells with improved resilience to inflammatory or profibrotic cues, thereby maximizing their therapeutic potential within the diseased lung [109].

Exosome‐based therapies provide another promising avenue, offering cell‐free systems capable of modulating immune and stromal interactions while overcoming the engraftment limitations of cell transplantation [110]. Additionally, human lung organoids and lung‐on‐chip platforms are emerging as powerful tools to dissect microenvironment–stem cell interactions with unprecedented physiological relevance [111, 112]. These platforms can model ECM alterations, immune–epithelial crosstalk, and mechanical forces, facilitating the discovery of microenvironment‐targeted combination strategies. Integrating stem cell–based approaches with approved antifibrotic agents such as pirfenidone and nintedanib may further enhance the consistency and durability of therapeutic outcomes [113, 114].

Progress in microenvironmental engineering, stem cell modification, and next‐generation disease models is likely to be key for translating microenvironment‐optimized stem cell therapies for PF into the clinic. Clarifying the bidirectional interactions between transplanted cells and the host lung niche will help guide the development of safer, more effective, and more durable regenerative strategies.

**Nomenclature**
Arp2/3: Actin‐related protein 2/3
AB: Activated basal cells
AEC2: Alveolar type II epithelial Cell
ARDS: Acute respiratory distress syndrome
AMs: Alveolar macrophages
ATII: Alveolar type II
bFGF: Basic fibroblast growth factor
BALF: Bronchoalveolar lavage fluid
CS: Cigarette smoke
JNK: c‐Jun N‐terminal kinase
CoQ10: Coenzyme Q10
CXCR4: CXC chemokine receptor 4
COX‐2: Cyclooxygenase‐2
CF: Cystic fibrosis
DOCK1: Dedicator of cytokinesis 1
ECM‐DTSs: ECM–modified decellularized tendon slices
ELMO1: Engulfment and cell motility protein 1
EMT: Epithelial–mesenchymal transition
ECM: Extracellular matrix
ERK: Extracellular signal‐regulated kinase
EVs: Extracellular vesicles
FGFs: Fibroblast growth factors
FF: Fibroblastic foci
GO: Gene Ontology
GBM: Glioblastoma
G‐actin: Globular actin
HSCs: Hematopoietic stem cells
HO‐1: Heme oxygenase‐1
hBMSCs: Human bone marrow‐derived MSCs
hMSCs: Human MSCs
HUVECs: Human umbilical vein endothelial cells
IPF: Idiopathic pulmonary fibrosis
iPSC‐MSCs: Induced pluripotent stem cell–derived MSCs
iPSCs: Induced pluripotent stem cells
Intgβ1: Integrin β1
IP‐10: Interferon‐gamma‐induced protein 10
IL‐1β: Interleukin‐1β
IL‐6: Interleukin‐6
ILDs: Interstitial lung diseases
KLF4: Kruppel‐like factor 4
MMPs: Matrix metalloproteinases
MT1‐MMP: Membrane‐type 1 MMP
MSC‐EVs: Mesenchymal stem cell–derived extracellular vesicles
MSC: Mesenchymal stem cells
MIF: Migration inhibitory factor
MAPK: Mitogen‐activated protein kinase
mESCs: Mouse embryonic stem cells
MPB: Multipotent basal cells
MVBs: Multivesicular bodies
MuSC: Muscle stem cell
m6A: N6‐methyladenosine
NK: Natural killer
Nrf2: Nuclear factor erythroid 2‐related factor 2
NF‐κB: Nuclear factor kappa‐light‐chain‐enhancer of activated B cells
PDLSCs: Periodontal ligament stem cells
PB: Proliferating basal cells
PGE2: Prostaglandin E2
PF: Pulmonary fibrosis
PUN: Punicalagin
Rac1: Ras‐related C3 botulinum toxin substrate 1
rBMSC: Rat bone marrow mesenchymal stem cell
rhHsp90α: Recombinant human heat shock protein 90‐alpha
SPB: Secretory‐primed basal cells
SRF: Serum response factor
SOX5: Sex‐determining region Y‐box protein 5
scRNA‐Seq: Single‐cell sequencing
SDF‐1: Stromal cell‐derived factor 1
TIMPs: Tissue inhibitors of metalloproteinases
TADs: Topologically associating domains
TAZ: Transcriptional coactivator with PDZ‐binding motif
TGF‐α: Transforming growth factor‐alpha
TGF‐β: Transforming growth factor‐β
TNF‐α: Tumor necrosis factor‐alpha
VEGF: Vascular endothelial growth factor
YAP: Yes‐associated protein.

## Disclosure

All the authors have read and approved the final version of the manuscript being submitted.

## Conflicts of Interest

The authors declare no conflicts of interest.

## Author Contributions

Zihan Zhou and Jie Huang conceptualized the ideas and wrote the manuscript. Zihan Zhou, Jie Huang, Shuhua Han, Yuanfang Duan, and Minqi Hong reviewed and revised the manuscript.

## Funding

This work was supported by the National Natural Science Foundation of China (Grant 82204004) and the Zhongda Hospital ‐ Lianyungang First People’s Hospital Joint Fund (Grant zdlyg28).

## Data Availability

Data sharing not applicable to this article as no datasets were generated or analyzed during the current study.
